# Inhibition of the NADPH Oxidase Pathway Reduces Ferroptosis during Septic Renal Injury in Diabetic Mice

**DOI:** 10.1155/2022/1193734

**Published:** 2022-02-27

**Authors:** Weifeng Yao, Haofeng Liao, Mengya Pang, Lijie Pan, Yu Guan, Xiaolei Huang, Ziqing Hei, Chenfang Luo, Mian Ge

**Affiliations:** ^1^Department of Anesthesiology, Third Affiliated Hospital, Sun Yat-sen University, Guangzhou, Guangdong 510630, China; ^2^Vaccine Research Institute of Sun Yat-sen University, Guangzhou 510630, China; ^3^Department of Anesthesiology, Shenzhen Maternity and Child Healthcare Hospital, Southern Medical University, Shenzhen 518116, China

## Abstract

**Background:**

Obesity and type 2 diabetes mellitus (DM) contribute to a higher mortality rate in patients with septic acute kidney injury (AKI) during sepsis. Reactive oxygen species (ROS) is the major injury factor for sepsis. This study was aimed at exploring the potential therapeutic drug for septic AKI targeting on ROS.

**Methods:**

A murine septic AKI model was established in both wild-type and high-fat diet-fed (HFD) mice. NADPH oxidase inhibitor Vas2870 was used *in vivo* to explore the role of NADPH oxidase in ROS release in septic AKI in diabetic mice. Ferrostatin-1 was administered to investigate the role of ferroptosis in ROS accumulation during NADPH oxidase activating in septic AKI in diabetic mice.

**Results:**

Compared to chow diet-fed mice, HFD diabetic mice which were subjected to LPS exhibited aggravated renal function (blood urea nitrogen, creatinine clearance, and serum cystatin C) and oxidative stress (malondialdehyde, 4-HNE, ROS, 8-OHdG, and NADPH oxidase), thus resulting in a higher mortality rate. Septic renal injury was significantly attenuated by the ferroptosis inhibitor Fer-1 in HFD-challenged mice. Furthermore, ferroptosis accumulation and related protein expression (ASCL4, FTH1, and GPX4) were altered by LPS stimulation in HFD-challenged mice and suppressed by NADPH oxidase inhibition via Vas2870 *in vivo*. In summary, NADPH inhibition restored septic renal function from injury by suppressing ferroptosis accumulation in HFD-challenged mice.

**Conclusion:**

These results suggest that targeting NADPH-mediated ROS release and ferroptosis accumulation is a novel therapeutic strategy to protect the kidney from septic injury in patients with obesity and type 2 DM.

## 1. Introduction

Diabetes mellitus (DM) is a prevalent global health problem that is one of the fastest growing emergencies of the 21st century, with approximately 10% morbidity [1]. Sepsis, another critical global medical condition, presents a mortality rate of approximately 26% [2]. Moreover, a recent study showed that approximately 21.8% of patients with sepsis had a medical history of DM [3]. Patients with DM show increased morbidity and mortality from sepsis compared to those in the nondiabetic population [4].

Acute kidney injury (AKI) is a common complication of sepsis, with a morbidity rate of 40% [5]. Although Esper et al. showed that diabetic patients were less likely to develop acute respiratory failure and hematological dysfunction than those without diabetes [6], recent studies indicate that diabetes is an independent risk factor for postoperative infection and sepsis-associated AKI [6, 7]. Sepsis-associated AKI results in higher mortality (20%–60%) compared with that of other types of AKI [8]. Reducing sepsis-associated AKI and restoring renal function can improve prognosis, particularly in patients with DM [9].

The pathogenesis of sepsis-associated AKI is poorly understood, and several factors, including systemic hemodynamics, renal microcirculation disturbance, and renal hypoxia, may be involved [10]. The production of reactive oxygen species (ROS) from the kidneys or other organs can directly damage renal parenchyma and may aggravate renal microvascular dysfunction, thus exacerbating already severe hypoxia and increasing ROS generation [11]. In diabetic patients, a high blood glucose level increases oxidative stress, leading to increased ROS accumulation and delayed recovery of sepsis-associated AKI [12]. Effective promotion of the clearance or inhibition of ROS production is an important strategy for the treatment of sepsis-associated AKI, particularly in patients with DM.

NADPH oxidases (NOXs) are recognized as the main source of ROS. NOXSs are membrane-spanning enzymes that produce hydrogen peroxide (NOX4, DUOX1-2) or superoxide (NOX1-3, NOX5) in an NADPH-dependent manner [13]. Nox2 and NOX4 are the main NOX subtypes that produce ROS in the kidneys [14–16]. A large accumulation of ROS leads to the formation of cellular ferroptosis. Ferroptosis, a type of programmed cell death, is characterized by iron-dependent accumulation of ROS, which can lead to organ dysfunction [17]. Inhibition of the NADPH oxidase pathway may provide a novel solution to ROS accumulation during sepsis-associated AKI.

The aim of the current study is to investigate the effect of NADPH oxidase inhibition on reduction of renal ROS release and subsequent ferroptosis accumulation and its ability to decrease sepsis mortality by restoring renal function in high-fat diet- (HFD-) challenged mice.

## 2. Methods and Materials

### 2.1. Animals and Ethics Statement

5-week-old male C57BL/6 mice were purchased from the Model Animal Research Center of Nanjing University and housed in the animal center barrier system (22 ± 2°C, 50 ± 5% humidity, and 12 h light/dark cycle) and provided with free access to water and food. Obesity was induced by feeding mice a high-fat diet (HFD) containing 60% fat calories (D12492; Research Diets, USA) for 12 weeks, while mice on a regular rodent chow diet (5% fat wt./wt.; Laboratory Animal Center of Sun Yat-sen University, China) were used as lean controls. Body weight was evaluated every 2 weeks, and daily food intake was monitored during the dietary intervention. The experimental animal protocol was approved by the Animal Ethics Committee of the Sun Yat-sen University. This committee is standardly guided by the Care and Use of Laboratory Animals (1996).

### 2.2. Glucose and Insulin Tolerance Tests

Glucose levels were measured at the indicated times with an Optium Xceed glucometer (Abbott Diabetes Care, Inc., Alameda, CA). For the oral gavage glucose tolerance test (OGTT), mice were fasted for 16 h overnight, then treated with intragastric glucose (2 g/kg wt.), and tail vein blood glucose levels were measured at 0, 30, 60, 120, and 180 min. For the intraperitoneal insulin tolerance test (ipITT), mice were fasted for 6 h and then injected intraperitoneally with insulin (0.65 U/kg wt.), and tail vein blood glucose levels were measured at 0, 30, 60, and 120 min.

### 2.3. LPS-Induced Septic Kidney Injury and Treatment

After dietary intervention, mice were subjected to various treatments. The mice were treated with a single dose of LPS (10 mg/kg, L2880, Sigma-Aldrich, Germany) or phosphate-buffered saline (PBS) via intraperitoneal injection.

For ferroptosis inhibition experiments, mice were injected intraperitoneally with ferrostatin-1 (10 mg/kg, HY-100579, MedChemExpress, USA) or vehicle 1 h before LPS administration. For NADPH oxidase inhibition, Vas2870 (10 mg/kg, HY-12804, MedChemExpress, USA) or vehicle was injected intraperitoneally 3 h before LPS administration. Twenty-four hours after LPS or PBS injection, mice were euthanized, and blood and kidneys were collected for further analysis.

### 2.4. Hematoxylin-Eosin Staining and Periodic Acid–Schiff Staining

Fresh kidney tissues were collected, fixed in 4% neutral formalin buffer solution (HT50-1-2; Sigma-Aldrich, Germany), and sectioned (5 *μ*m). Hematoxylin–eosin (HE) staining was used for histopathological analysis using a light microscope (Leica Corporation, Germany). Tubular injuries were graded by a score of 0–4 (0, no change; 1, change affecting <25% of the field; 2, change affecting 25%–50% of the field; 3, change affecting 50%–75% of the field; and 4, change affecting >75% of the field). Glycogen storage in kidney tissue was analyzed with a periodic acid–Schiff staining kit (G1008, Servicebio, China) following the manufacturer's instructions. Other fresh tissues were put into liquid nitrogen and sliced. The fat content of renal tubular epithelial cells was analyzed using the BODIPY staining kit (D3922, Thermo, USA) according to the manufacturer's instructions.

### 2.5. Serum Urea Nitrogen (BUN), Creatinine (CCr), Cystatin C (Cysc), and Glutathione Reductase (GR) Measurement

BUN, CCr, Cysc, and GR levels were analyzed by the automatic biochemistry analyzer (Watford Olympus AU640, United Kingdom).

### 2.6. ROS Measurement

The ROS levels in the kidney were detected using a DCFH-DA assay kit (BB-470536, Best Bio, China). After washing with cleaning solution at room temperature, the tissues were incubated with the DCFH-DA staining [18] solution for 1 h at 37°C, and images were captured using a fluorescence microscope (EVOS FL, Life Technology, USA).

### 2.7. Malondialdehyde (MDA) Level Measurement

MDA levels were measured using an MDA-specific assay reagent kit (A003-1-1, Jiancheng, China) according to the manufacturer's instructions, and the absorbance was measured at 532 nm using a microplate reader (ELX800, Bio-Tek, USA). The protein concentration of each sample was measured using a BCA Protein Assay kit (BCA-23225, Thermo Fisher Scientific, USA).

### 2.8. Transmission Electron Microscopy (TEM)

Kidney tissues were fixed with 4% glutaraldehyde solution (G1102, Servicebio, China) for 12 h, stained with 1% osmium tetroxide (18459, Ted Pella Inc., USA), dehydrated with acetone, and embedded in resin. Samples were then sectioned (60 nm), mounted on copper grids, stained, and examined using TEM (HT7700, Hitachi, Japan).

### 2.9. Immunohistochemistry Staining

After dewaxing and dehydration, thin sections (5 *μ*m) of kidney tissues were deparaffinized, blocked, and incubated with anti-4-HNE (1 : 200; ab46545, Abcam Technology, United Kingdom), anti-8-OHDG (1 : 800; ab48508, Abcam Technology, United Kingdom), and anti-GPX4 (1 : 200; ab125066, Abcam Technology, United Kingdom) 12 h at 4°C. Sections were stained with an HRP IHC kit (GK500710, Gene Tech, China) according to the manufacturer's instructions. Staining intensity was observed under a light microscope (Leica Corporation, Germany) and analyzed using ImageJ software (National Institutes of Health, USA). Finally, ImageJ (V1.8.0.112, National Institutes of Health, USA) was used to analyze the positive area in each slide and make statistics.

### 2.10. Western Blotting

Mouse kidneys were homogenized and centrifuged at 13000 rpm at 4°C for 30 minutes to take the supernatant. Extracts of the kidney tissue were prepared in lysis buffer. The total protein was separated on 5-20% acrylamide gel by using twelve alkyl sulfate polyacrylamide gel electrophoresis (SDS-PAGE) and then transferred to polyvinyl fluoride two (PVDF). After sealing with 5% fat-free milk for 1 hour, the membrane was incubated with the corresponding primary anti-rabbit antibody at 4°C overnight. Whole-cell lysates of the kidney were analyzed using western blotting to detect the expression of NOX2, NOX4, ACSL4, GPX4, FTH1, and *β*-actin. Primary antibodies against Gp91phox/NOX2 (1 : 1000; ab129068, Abcam Technology, United Kingdom), NOX4 (1 : 1000; ab133303, Abcam Technology, United Kingdom), ACSL4 (1 : 1000; A6826, ABclonal Technology, China), GPX4 (1 : 1500; ab125066, Abcam Technology, United Kingdom), FTH1 (1 : 1000; ab75972, Abcam Technology, United Kingdom), and *β*-actin (1 : 10000; AC026, ABclonal Technology, China) were used. The imprinting was visualized by enhanced chemiluminescence (ECL) system, and the gray value was scanned and quantified by ImageJ software.

### 2.11. Statistical Analysis

All data were analyzed using SPSS version 22.0. Quantitative data are presented as the mean ± SEM from multiple samples (*n* = 6 − 10 for each group). The Kolmogorov-Smirnov test was used to test the normality of the data, and Levene's test was used to test the homogeneity of variance. The *t*-test was used to analyze the differences between two groups, followed by the LSD post hoc test. Comparisons of multiple groups were analyzed using two-way repeated measures analysis of variance (ANOVA) or one-way ANOVA. Differences were considered statistically significant when *p* values were less than 0.05.

## 3. Results

### 3.1. High-Fat Diet-Induced Diabetes Aggravated Septic Renal Injury

**Figure 1:**
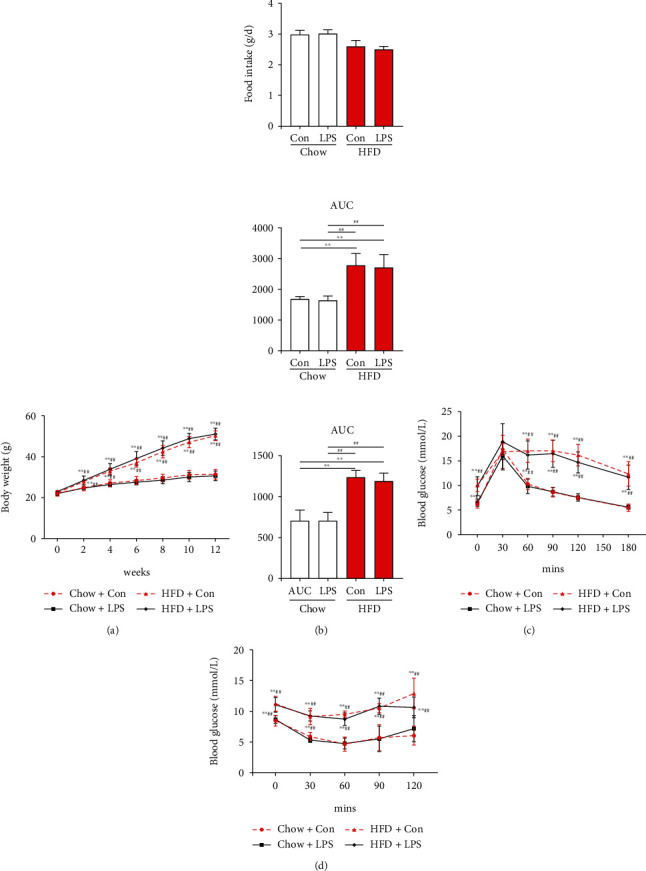
Wild-type mice were challenged with a high-fat diet (HFD) for 12 weeks to induce diabetes. (a) Body weight (BW) gain over time. (b) Food intake was monitored daily for 3 d, and average daily food intake (g) was calculated. (c) Glucose tolerance test (GTT). The total insulin secretion (AUC) is calculated from (c). (d) Insulin tolerance test (ITT) and area under the curve. Each bar represents the mean ± SEM (*n* = 6 − 8). Compared with the chow+con group, ^∗^*p* < 0.05 and ^∗∗^*p* < 0.01. Compared with the chow+LPS group, ^#^*p* < 0.05 and ^##^*p* < 0.01. One-way ANOVA with Tukey's test. Chow+con: mice on 12 weeks of the chow diet received the same volume of solvent without LPS; HDF+con: mice on 12 weeks of the HDF diet received the same volume of solvent without LPS; chow+LPS: mice on 12 weeks of the chow diet subjected to LPS (10 mg/kg, intraperitoneally) stimulation; HDF+LPS: mice on 12 weeks of the HDF diet subjected to LPS (10 mg/kg, intraperitoneally) stimulation.

From the second week of HFD intervention, a significant increase in body weight was observed in the HFD group (*p* < 0.01), which persisted until the end of intervention (Figure 1(a)). No differences in food intake were observed between the HFD and chow-diet groups (Figure 1(b)). The GTT results showed deteriorated glucose intolerance in HFD-challenged mice with a significantly larger AUC value (Figure 1(c)). Additionally, higher blood glucose levels at time points 0, 30, 60, 90, and 120 min with a higher AUC value were observed in HFD-challenged mice during the ITT (Figure 1(d)).

**Figure 2:**
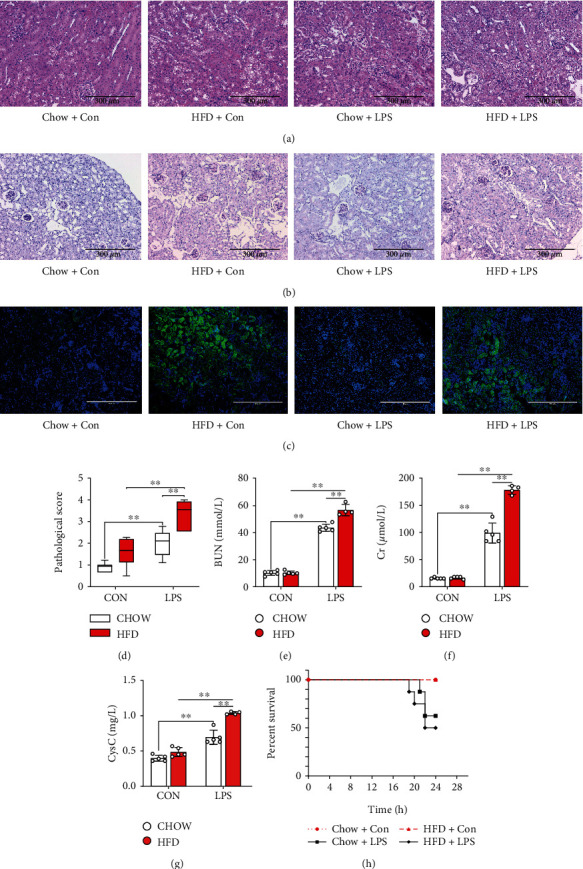
Diabetes-aggravated septic renal injury in mice. Renal pathology was clearly observed via renal H&E ((a) 200x) and periodic acid-Schiff (PAS) staining ((b) 200x). BODIPY staining ((c) 200x) was used to analyze the phospholipid accumulation in the proximal tubular cells of different groups of mice. The pathological score (d) was evaluated according to renal H&E staining. Serum urea nitrogen (BUN) (e), creatinine (CCr) (f), and cystatin C (Cysc) (g) levels, which reflect renal function, were determined. Furthermore, the survival rate (h) of diabetic mice subjected to LPS was calculated. Each bar represents the mean ± SEM (*n* = 4 − 8). ^∗^*p* < 0.05 and ^∗∗^*p* < 0.01, one-way ANOVA with Tukey's test.

The results of H&E (Figures 2(a) and 2(b)) showed that high-fat diet-induced diabetes aggravated septic renal injury and caused fat accumulation in the proximal tubular cells (Figure 2(c)), which had higher injury scores (Figure 2(d)) in HFD-challenged mice than those in chow diet-fed mice when subjected to LPS stimulation. Moreover, the serum BUN (Figure 2(e)), CCr (Figure 2(f)), and Cysc (Figure 2(g)) levels, which reflect renal function, were significantly increased in HFD-challenged mice compared with those in chow diet-fed mice when subjected to LPS stimulation. LPS stimulation led to higher motility in HFD-challenged mice than in chow-fed mice. As shown in Figure 2(h), the LPS stimulation significantly decreased the survival rate of mice which was lower in HFD-challenged mice than those in chow diet-fed mice.

### 3.2. NADPH Oxidase and Ferroptosis Were Activated during Septic Renal Injury in HFD-Challenged Diabetic Mice

**Figure 3:**
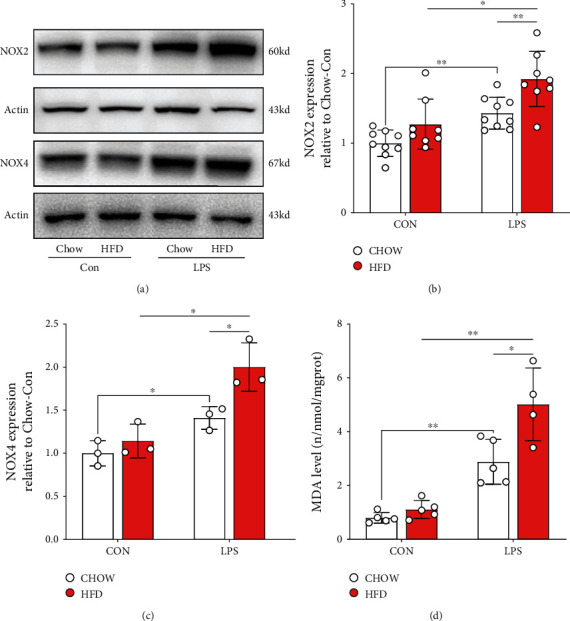
Renal NADPH oxidase subunits were activated in diabetic mice subjected to LPS. Renal NADPH subunits, NOX2 and NOX4, were detected by western blotting (a). Gray analysis was performed according to the bands of NOX2 (b) and NOX4 (c). Renal malondialdehyde (MDA) (d) expression was measured using enzyme-linked immunosorbent assay (ELISA). Each bar represents the mean ± SEM (*n* = 3 − 8). ^∗^*p* < 0.05 and ^∗∗^*p* < 0.01, one-way ANOVA with Tukey's test.

**Figure 4:**
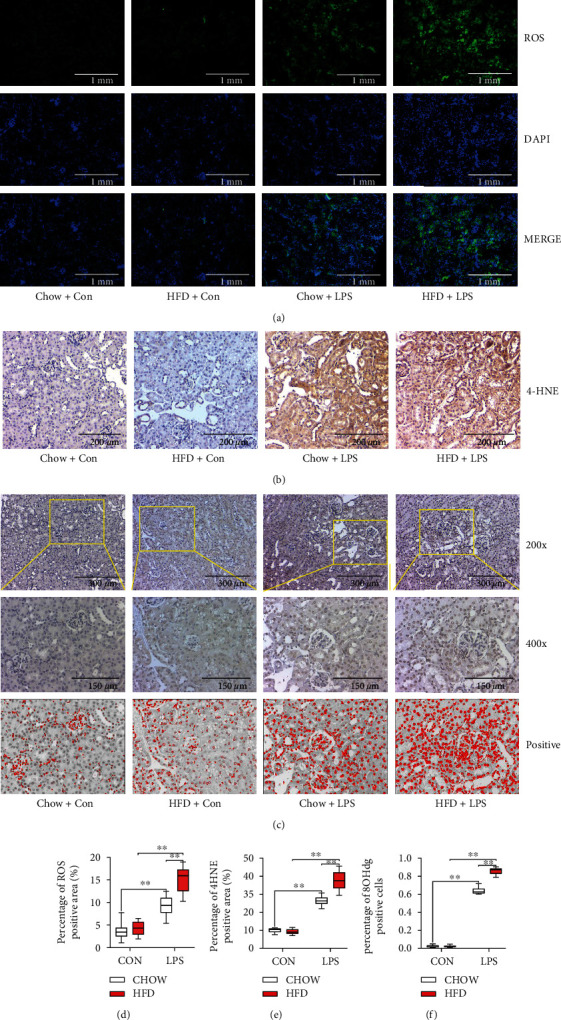
Diabetes-exacerbated oxidative stress during septic renal injury. Renal reactive oxygen species (ROS) levels were detected by immunofluorescence (a). Renal 4-hydroxynonenal (4-HNE) immunohistochemical staining (b) and 8-hydroxydeoxyguanosine (8-OHdG) immunohistochemical staining (c) were performed to evaluate oxidative stress levels in the kidneys. Semiquantitative analysis of ROS immunofluorescence staining (d), 4-HNE immunohistochemical staining (e), and 8-OHdG immunohistochemical staining (f). Each bar represents the mean ± SEM (*n* = 6 − 8). ^∗^*p* < 0.05 and ^∗∗^*p* < 0.01, one-way ANOVA with Tukey's test.

To investigate the underlying mechanism of HFD-challenged diabetes-deteriorated septic renal injury, NADPH oxidase activation and changes in ferroptosis accumulation were measured. As shown in Figures 3(a)–3(c), levels of NADPH oxidase subunits, NOX2 and NOX4, significantly increased in HFD-challenged mice compared to those in chow-fed mice when subjected to LPS stimulation. Additionally, LPS stimulation led to higher renal MDA (Figure 3(d)), ROS (Figure 4(a), Figure 4(d)), 4-HNE (Figure 4(b), Figure 4(e)), and 8-OHdG expression levels (Figures 4(c) and 4(f)) in chow-fed mice. HFD-induced diabetes can aggravate renal oxidative stress via NADPH oxidase subunit activation.

**Figure 5:**
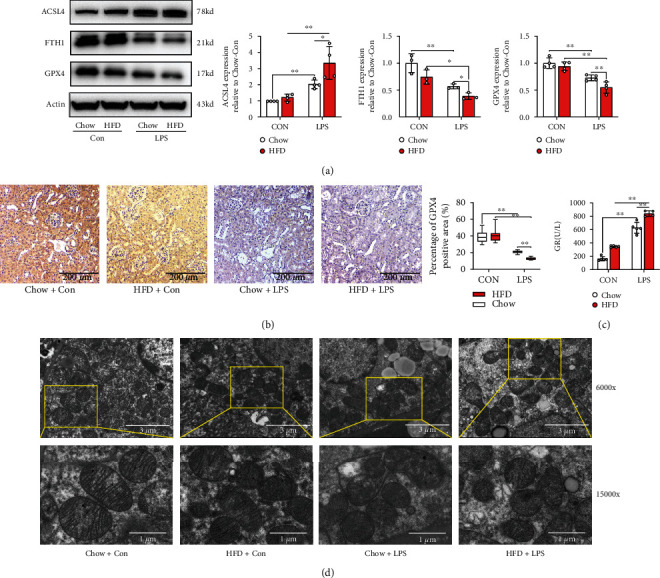
Renal ferroptosis accumulated during septic renal injury in diabetic mice. The expression levels of ferroptosis-related proteins including ASCL4, FTH1, and GPX4 were determined by western blotting (a). GPX4 levels were also detected by immunohistochemical staining (b). The renal scavenging effect on ROS is reflected by the glutathione reductase (GR) level (c). Morphological features of mitochondria were measured using electron microscopy (d). Each bar represents the mean ± SEM (*n* = 3 − 6). ^∗^*p* < 0.05 and ^∗∗^*p* < 0.01, one-way ANOVA with Tukey's test.

As ROS accumulation leads to ferroptosis, we detected ferroptosis in renal sepsis. As shown in Figures 5(a) and 5(b), ferroptosis-related protein ASCL4 significantly increased in LPS-stimulated groups, and ASCL4 expression was higher in HFD-challenged mice than that in chow-fed mice when subjected to LPS stimulation. Additionally, a decrease in the levels of ferroptosis-related proteins FTH1 and GPX4 was observed in HFD-challenged mice subjected to LPS stimulation (*p* < 0.01). Simultaneously, glutathione reductase (GR) level (Figure 5(c)), which is involved in the formation of ferroptosis by regulating glutathione (GSH) generation through the NADPH pathway, was significantly increased in HFD-challenged mice than in chow-fed mice when subjected to LPS stimulation. To verify this observation, ferroptosis in renal tubular epithelial cell mitochondria was detected by electron microscopy (Figure 5(d)). We found that the cell membrane was broken and vacuolated, mitochondrial spine was decreased or absent, and membrane density was increased under LPS stimulation, which was deteriorated in HFD-challenged mice.

### 3.3. Ferroptosis Inhibitor Reversed Aggravated Septic Renal Injury in HFD-Challenged Mice

**Figure 6:**
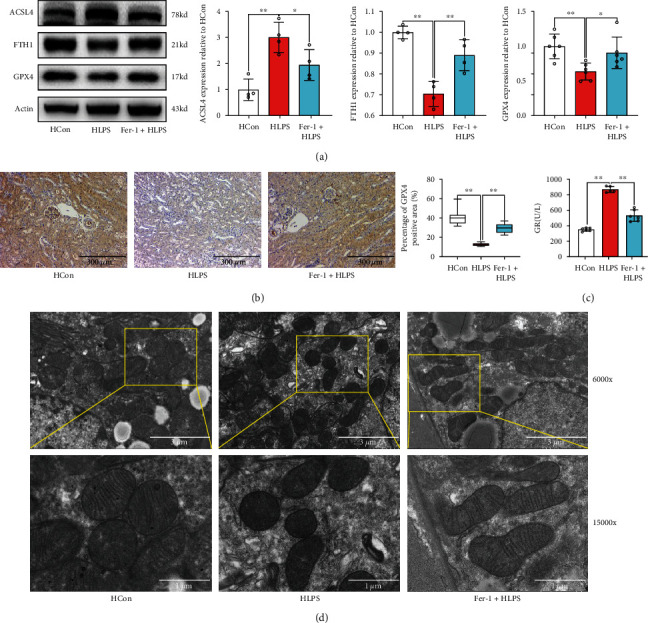
Fer-1 effectively inhibited ferroptosis accumulation during septic renal injury in diabetic mice. The expression levels of ferroptosis-related proteins, including ASCL4, FTH1, and GPX4, were determined by western blotting (a). GPX4 levels were also detected by immunohistochemical staining (b). The GR level is indicative of the renal scavenging effect on ROS (c). Morphological features of mitochondria were measured using electron microscopy (d). Each bar represents the mean ± SEM (*n* = 3 − 8). ^∗^*p* < 0.05 and ^∗∗^*p* < 0.01, one-way ANOVA with Tukey's test. Hcon: mice fed a HDF diet for 12 weeks received the same volume of solvent without LPS; HLPS: mice fed a HDF diet for 12 weeks and subjected to LPS (10 mg/kg, intraperitoneally) stimulation; Fer-1+HLPS: ferrostatin-1 (Fer-1) (10 mg/kg) was administered intraperitoneally 1 h before LPS administration.

To investigate the role of ferroptosis in septic renal injury in HFD-challenged mice, the ferroptosis inhibitor ferrostatin-1 (Fer-1) was used *in vivo*. As shown in Figures 6(a) and 6(b), ferroptosis accumulation induced by LPS stimulation in HFD-challenged mice was inhibited by Fer-1, as evidenced by an increase in ASCL4 protein expression and a decrease in FTH1 and GPX4 proteins. However, we found that there were no significant differences in NOX protein expressions between the Fer-1-treated groups and LPS-treated HFD diabetic mice (Supplemental Figure 1). Additionally, the expression of GR was significantly decreased by Fer-1 in the HLPS group compared to that in the HFD-challenged mice under LPS stimulation (Figure 6(c)). Moreover, the mitochondrial changes induced by LPS stimulation in HFD-challenged mice were restored after Fer-1 treatment (Figure 6(d)).

**Figure 7:**
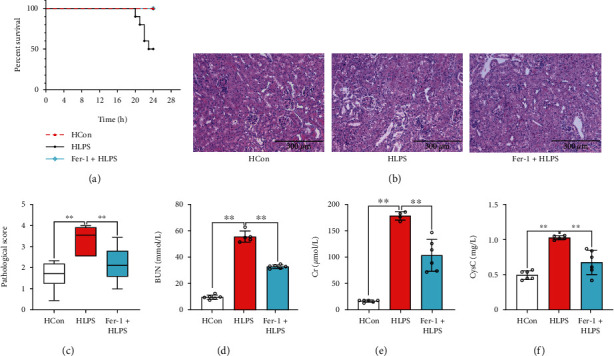
Inhibition of ferroptosis reduced septic renal injury. The survival rate (a) of the mice was calculated. Renal pathology was observed via renal H&E staining ((b) 200x). The pathological score (c) was evaluated according to renal H&E staining. Serum BUN (d), CCr (e), and Cysc (f) levels were determined. Each bar represents the mean ± SEM (*n* = 6 − 10). ^∗^*p* < 0.05 and ^∗∗^*p* < 0.01, one-way ANOVA with Tukey's test.

Thereafter, we investigated the effects of Fer-1 on renal pathology and function. As shown in Figure 7(a), Fer-1 treatment effectively increased the survival rate of HFD-challenged mice subjected to LPS stimulation. Moreover, the pathology (Figure 7(b)) and renal function, which were impaired during LPS stimulation, were restored after Fer-1 treatment in HFD-challenged mice, as evidenced by decreased pathological scores (Figure 7(c)) and expression levels of serum BUN (Figure 7(d)), CCr (Figure 7(e)), and Cysc (Figure 7(f)).

### 3.4. NADPH Inhibition Ameliorated Septic Renal Injury via Suppressing Ferroptosis Accumulation in HFD-Challenged Mice

**Figure 8:**
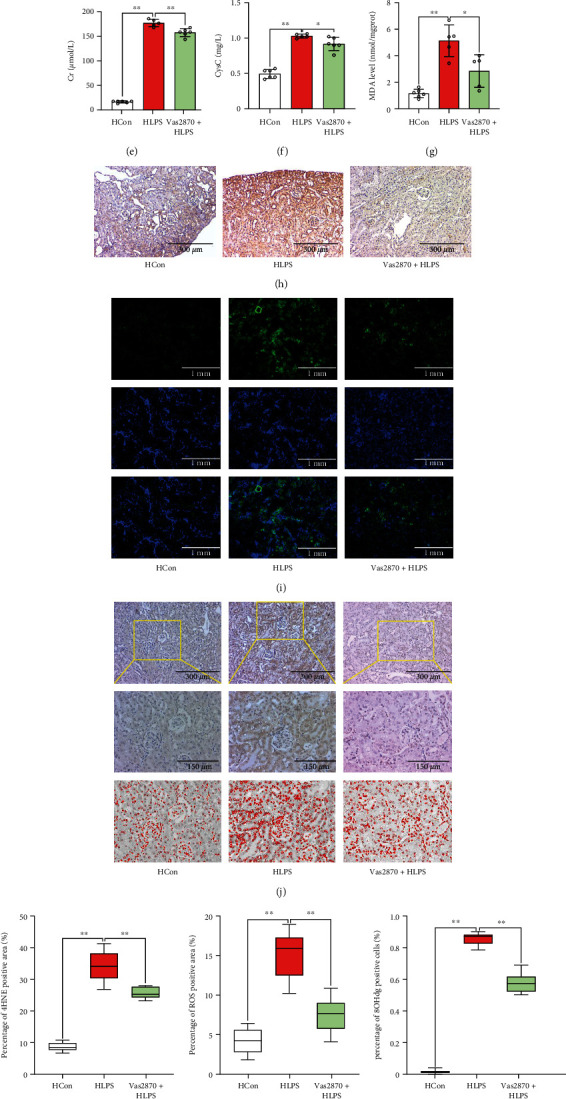
Inhibition of the NADPH oxidase pathway reduced septic renal injury via suppressing renal oxidative stress in diabetic mice. The survival rate (a) of the mice was calculated. Renal pathology was clearly observed via renal H&E ((b) 200x) staining and PAS staining ((c) 200x). Serum BUN (d), CCr (e), and Cysc (f) levels were determined. MDA was measured using ELISA (g). Renal 4-hydroxynonenal (4-HNE) immunohistochemical staining (h), renal ROS immunofluorescence staining (i), and 8-hydroxydeoxyguanosine (8-OHdG) immunohistochemical staining (j) were performed. Semiquantitative analysis of 4-HNE immunohistochemical staining (k), ROS immunofluorescence staining (l), and 8-OHdG immunohistochemical staining (m). Renal NADPH subunits NOX2 and NOX4 were detected by western blotting (n). Each bar represents the mean ± SEM (*n* = 4 − 10). ^∗^*p* < 0.05 and ^∗∗^*p* < 0.01, one-way ANOVA with Tukey's test. Hcon: mice fed a HDF diet for 12 weeks received the same volume of solvent without LPS; HLPS: mice fed a HDF diet for 12 weeks and subjected to LPS (10 mg/kg, intraperitoneally) stimulation; Vas2870+HLPS: Vas2870 (10 mg/kg) was administered intraperitoneally 3 h before LPS administration.

As ROS accumulation leads to ferroptosis and NADPH oxidase plays an important role in ROS generation, we used Vas2870, to investigate the restoration of renal function by inhibition of NADPH oxidase, which suppresses ferroptosis accumulation. As shown in Figure 8(a), inhibition of NADPH oxidase by Vas2870 increased the survival rate of HFD-challenged mice subjected to LPS stimulation. Moreover, the pathology (Figures 8(b) and 8(c)) and renal function, which were impaired during LPS stimulation, were restored after Vas2870 treatment in HFD-challenged mice, as evidenced by lower levels of serum BUN (Figure 8(d)), CCr (Figure 8(e)), and Cysc (Figure 8(f)).

Compared to HFD-challenged mice treated with solvent, NADPH oxidase inhibition resulted in a significant decrease in renal oxidative stress, as evidenced by lower MDA (*p* < 0.05) (Figure 8(g)), 4-HNE (*p* < 0.05) (Figure 8(h), Figure 8(k)), ROS generation (*p* < 0.05) (Figure 8(i), Figure 8(l)), and 8-OHdG (*p* < 0.05) (Figures 8(j) and 8(m)) expression levels in HFD-challenged mice subjected to LPS stimulation. The expressions of NADPH oxidase subunits, NOX2 and NOX4, which were activated by LPS stimulation, were significantly decreased after Vas2870 treatment in HFD-challenged mice (Figure 8(n)).

**Figure 9:**
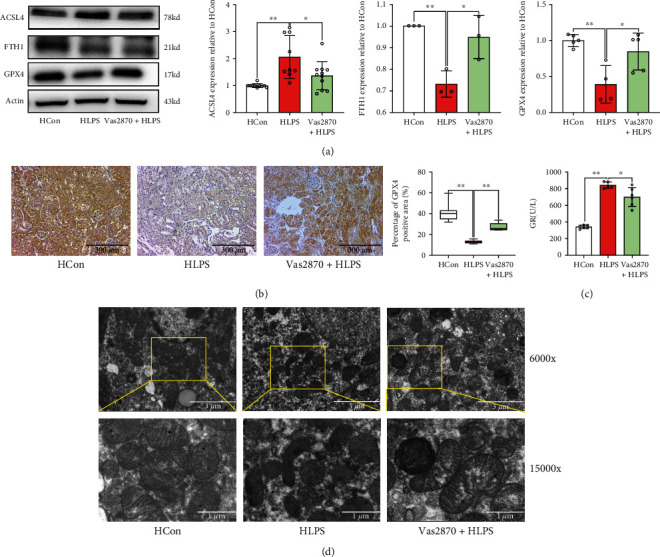
Inhibition of the NADPH oxidase pathway attenuated renal ferroptosis accumulation. Ferroptosis-related proteins, including ASCL4, FTH1, and GPX4, were determined by western blotting (a). GPX4 levels were also detected by immunohistochemical staining (b). The renal scavenging effect on ROS is reflected by the glutathione reductase (GR) level (c). Morphological features of mitochondria were measured using electron microscopy (d). Each bar represents the mean ± SEM (*n* = 3 − 8). ^∗^*p* < 0.05 and ^∗∗^*p* < 0.01, one-way ANOVA with Tukey's test.

Furthermore, the results of ferroptosis accumulation (Figures 9(a) and 9(b)) showed that ASCL4 protein expression increased, whereas FTH1 and GPX4 protein expression decreased after Vas2870 treatment in HFD-challenged mice subjected to LPS stimulation. This was accompanied by a significant decrease in GR levels after Vas2870 application in the HLPS group compared to that in the HFD-challenged mice under LPS stimulation (*p* < 0.05) (Figure 9(c)). Additionally, mitochondrial changes induced by LPS stimulation were restored by Vas2870 in HFD-challenged mice (Figure 9(d)). These results indicated that NADPH inhibition could restore septic renal function from injury by suppressing ferroptosis accumulation in HFD-challenged mice.

## 4. Discussion

The effect of obesity or type 2 diabetes on the survival of sepsis-associated AKI remains a controversial topic [19, 20]. In the current study, mice subjected to 12 weeks of a HDF diet were successfully obtained to mimic type 2 diabetes. We found that HFD-induced diabetes aggravated renal function and oxidative stress in mice subjected to LPS stimulation, which led to a higher mortality rate compared to that of chow-fed mice. Septic renal injury was attenuated by a ferroptosis inhibitor in HFD-challenged mice. Furthermore, ferroptosis accumulation, which was altered by LPS stimulation in HFD-challenged mice, was suppressed by the NADPH oxidase inhibitor, Vas2870, *in vivo*. Our results indicated that HFD-induced diabetes may act as a potential risk factor that aggravates renal injury, particularly under critical conditions such as sepsis.

Ferroptosis is a type of iron-dependent programmed cell necrosis, which is mainly caused by ROS accumulation and characterized by extensive lipid peroxidation and subsequent severe mitochondrial and cell membrane dysfunction [21, 22]. During AKI, renal tubular epithelial cell necroptosis [23], necrosis [24], and apoptosis [25] have been identified as the predominant types of cell death that lead to irreversible renal dysfunction. However, a recent study showed that ferroptosis is part of an adaptive response that unsuccessfully attempts to restore renal cellular homeostasis [26]; hence, pharmacological intervention is required to restore renal function [26], without inducing tubular epithelial cell necrosis or apoptosis. In the present study, ferroptosis inhibitor Fer-1 intervention reversed sepsis-associated AKI, which was aggravated in HFD-challenged mice. Our results indicate that it is critical to block ferroptosis formation in the early stage of sepsis-associated AKI, which may restore renal function by rebalancing renal cellular homeostasis. Our findings were consistent with those of Martin-Sanchez et al., in which Fer-1 could preserve renal function from nephrotoxic folic acid-induced AKI [27].

Notably, we found that ferroptosis was increased in HFD-challenged mice subjected to LPS stimulation. To date, studies have reported that ferroptosis can be activated in diabetes [28] and LPS stimulation [29]. However, in the present study, we found that renal ferroptosis elicited no significant effect in HFD-challenged mice without LPS stimulation. Therefore, in the process of sepsis-associated AKI in HFD-challenged mice, LPS stimulation initially induces ferroptosis, while the diabetes state may slow down ROS clearance and ferroptosis elimination, which together deteriorates renal function. Inhibition of ROS release may effectively reduce ferroptosis formation [30, 31]. NADPH oxidase is the main source of ROS production in patients with diabetes [32] and sepsis [33]. In the present study, Vas2870 effectively reduced ROS release and subsequently attenuated renal ferroptosis formation, resulting in the restoration of renal function following LPS stimulation in HFD-challenged mice. These results indicate that inhibition of NADPH oxidase may either reduce ROS release from LPS stimulation or decrease oxidative stress in the diabetic state, which synergistically attenuates ferroptosis formation and improves renal function.

Notably, in the current study, we only observed the change of mitochondria in renal tubular epithelial cells, although there are several other cell types including Sertoli, glomerular vascular endothelial, and immune cells that contribute to sepsis-associated AKI; therefore, the specific cell type undergoing ferroptosis during sepsis-associated AKI requires further investigation. And more molecular mechanisms will be found in our further study. Moreover, the NADPH oxidase pathway in addition to the mitochondrial enzymes associated with the respiratory chain promotes extracellular ROS generation. However, potential renal-protective effects of mitochondrial enzymes associated with the respiratory chain require further investigation.

## 5. Conclusion

HFD-induced diabetes exacerbates sepsis-associated AKI via upregulation of NADPH oxidase, subsequent ROS release, and ferroptosis accumulation. NADPH inhibition could restore septic renal function from injury by suppressing ferroptosis accumulation in HFD-challenged mice, suggesting that targeting NADPH-mediated ROS release and ferroptosis accumulation is a novel therapeutic strategy to protect the kidney from septic injury in patients with obesity and type 2 DM.

## Data Availability

The data that support the findings of this study are available from the corresponding author upon reasonable request.

## Abbreviations

HDF: High-fat diet
Fer-1: Ferrostatin-1
ROS: Reactive oxygen species
AKI: Acute kidney injury
NOXs: Nicotinamide adenine dinucleotide phosphate oxidase
DM: Diabetes mellitus
OGTT: Oral gavage glucose tolerance test
ipITT: Intraperitoneal insulin tolerance test
BUN: Serum urea nitrogen
CCr: Creatinine
GR: Glutathione reductase
Cysc: Cystatin C
MDA: Malondialdehyde
TEM: Transmission electron microscopy
HE: Hematoxylin–eosin
PAS: Periodic acid-Schiff.

## Supplementary Materials

Supplementary Materials Supplementary Figure 1: Fer-1 did not affect the protein expression of NOX in LPS-tread diabetic mice. The expression levels of NOX2 and NOX4 proteins were determined by western blotting (A). Each bar represents the mean ± SEM (*n* = 4). *ns* means *p* > 0.05, *T*-test.
