# Supplementary material for: Inhibition of the NADPH Oxidase Pathway Reduces Ferroptosis during Septic Renal Injury in Diabetic Mice
Source: Oxid Med Cell Longev. 2022 Feb 27;2022:1193734. doi: 10.1155/2022/1193734 (PMC8898803; doi:10.1155/2022/1193734)
Supplement: Supplementary Materials — Supplementary Figure 1: Fer-1 did not affect the protein expression of NOX in LPS-tread diabetic mice. The expression levels of NOX2 and NOX4 proteins were determined by western blotting (A). Each bar represents the mean ± SEM (n = 4). ns means p > 0.05, T-test. [file 1193734.f1.docx]

**Inhibition of the NADPH oxidase pathway reduces ferroptosis during septic renal injury in diabetic mice**

Weifeng Yao^1^, Haofeng Liao^1^, Mengya Pang^1^, Lijie Pan^2^, Yu Guan^1^, Xiaolei Huang^3^, Ziqing Hei^1^, Chenfang Luo^1^, Mian Ge^1^.

Supplemental Figure 1


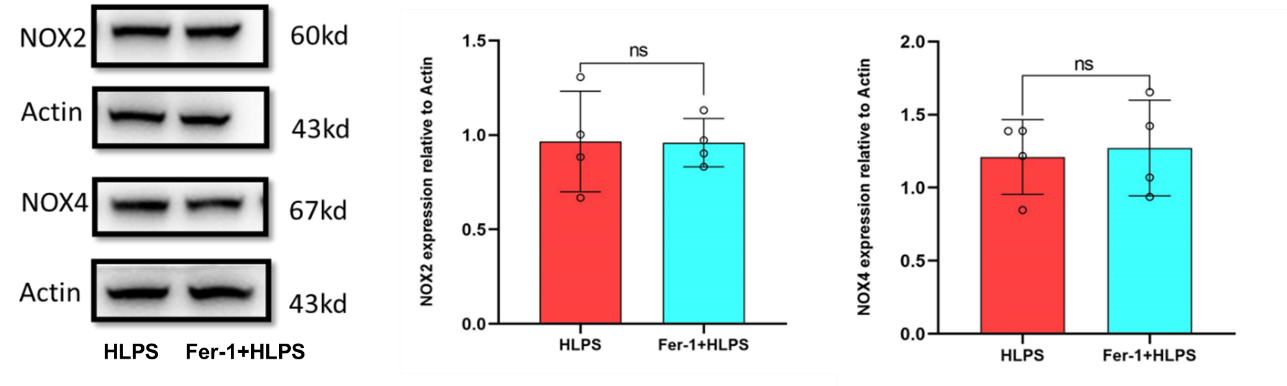


**Figure 1 The Fer-1 did not affect the protein expression of NOX in LPS-tread diabetic mice.**

The expression levels of NOX2 and NOX4 proteins were determined by western blotting (A). Each bar represents the mean ± SEM (*n = 4*). *ns* means *p* > 0.05, T-test.
